# In regards to Bogue J, Wan J, Lavey RS, Parsai EI. Dosimetric comparison of VMAT with integrated skin flash to 3D field‐in‐field tangents for left breast irradiation. Journal of applied clinical medical physics. 2019 Feb;20(2):24‐9

**DOI:** 10.1002/acm2.12607

**Published:** 2019-05-14

**Authors:** Biplab Sarkar, Tharmarnadar Ganesh, Anusheel Munshi, Kanika Bansal, Khushboo Rastogi, Sandeep Tiwari, Arjunan Manikandan, Bidhu Kalyan Mohanti

**Affiliations:** ^1^ Department of Radiation Oncology Manipal Hospitals New Delhi India; ^2^ Department of Radiation Oncology, Apollo Proton Cancer Centre Chennai TN India

Dear Editor

We have read the article published by Bogue J *et al*.^1^ and in conjunction the few other articles^2^, ^3^, ^4^ published in your esteemed journal in recent times. These articles are related to the use of volumetric modulated arc therapy (VMAT) technique for breast and chest wall radiotherapy. We hereby raise two concerns regarding the technique by Bogue J *et al*., and other authors^2^, ^3^, ^4^, ^5^, ^6^ which have more to do with the planning systems and their optimization processes rather than about the authors or their methodologies.

Our first concern is about the use of large arc angles and the second concern relates to the practice of using virtual bolus for the creation of flash margin. A short literature survey on the arc angles used in breast irradiation and associated skin flash margins is justified.

Regarding the arc angle, literature survey shows that Bogue et *al*., Byren et *al*., Rossi et *al*., Tyren et *al*. and Balaji et *al*.^1^, ^2^, ^3^, ^4^, ^5^ respectively used four arcs of 240°, two arcs of 240°, two arcs of 190° and two arcs of 240°, two arcs of 240° and two arcs of 190°. Although VMAT‐based breast/chest wall radiotherapy is getting popular in recent time, the conventional parallel‐opposing tangential beam 3DCRT (three dimensional conformal radiotherapy) technique still remains as the universally accepted standard technique being followed by a majority of users irrespective of the planning system and linear accelerator. While adopting the VMAT technique for this site, the fixed‐gantry tangential pair of beams is replaced by arcs ranging between 190° and 240°. A VMAT‐based breast and chest wall radiotherapy technique was described by Popescu *et al*. and Nicolini *et al*. for Eclipse planning system (Varian Medical System, Polo Alto, CA, USA) using an arc angle of 190° and 243° ± 6°, respectively.^7^, ^8^ While planners have typically employed large arc lengths in replacement of tangential fixed beams in Eclipse^1^, ^3^, ^5^, ^6^, ^7^, ^8^ and RayStation^2^, ^4^ (Ray Search Laboratories, Stockholm, Sweden), there have been successful examples of planners using short tangential arcs in other systems like Monaco (CMS Elekta, Sunnyvale, CA, USA) to produce a clinically acceptable dose distribution.^9^, ^10^, ^11^ Thus, the advantage of shorter arc lengths to reduce the normal tissue irradiation volume should appear as logical and attractive. The planners who use large arc lengths in VMAT‐based breast/chest wall radiotherapy in some of the planning systems may need to take a closer look at the optimizer in those systems in order to efficiently produce clinically acceptable dose distribution with shorter arc lengths. One cannot rush to say that Eclipse/RayStation users always showed a tendency to use large arc lengths, whereas Monaco users were able to generate good plans with shorter tangential arcs. Nevertheless, we could not find any head‐on comparison between different planning systems exploring the interplay between VMAT arc angle chosen and the acceptability of dose distribution. The more likely reason points to the sub‐optimal performance of the optimizer to produce a clinically acceptable dose distribution.

Our personal experience with Eclipse also substantiates the second reason. Even eclipse optimizer does not work well with an arc length less than 30°. As described by Munshi et al., Giri et.al and Virén et.al., Monaco works well with shorter arcs.^9^, ^10^, ^11^ Munshi et al., further established the geometrical relationship between the 3DCRT beam and VMAT arc angle for breast/chest wall radiotherapy.^9^ Therefore, it is more geometrically correct to replace the conventional tangential beam with a VMAT beam having shorter arc length in order to account for and maintain the solid geometrical equivalence between the two techniques. However not all planning systems are capable of producing clinically acceptable dose distributions with shorter arcs.^1–8^ Use of large arcs will make dose statistics for planning target volume better (dose coverage, heterogeneity index, hot spot) but will completely change the low dose characteristics of the treatment plan. Changes in low dose characteristics of the treatment plan is highly undesirable since it would make the clinicians uncomfortable with difficult choices for dose limits of critical organs (lung, heart)^9^ and have its own demerits and implications.^10^, ^11^

The other point is the generation of the skin flash margin using a virtual bolus for Eclipse planning system.^1^, ^2^, ^3^, ^4^, ^6^, ^7^, ^8^ Use of flash margin is an important requirement in the breast and chest wall radiotherapy and is required for all types of delivery techniques including free breathing, deep inspiration breath hold, and gated delivery conditions. Although skin flash tool is an available feature in Eclipse IMRT optimizer, it is not available in VMAT mode making it inferior to VMAT optimizers of other planning systems like Monaco and RayStation. The present form of the creation of flash margin using a virtual bolus is not the ideal way to create it.

In our submission, planning systems should be able to generate a clinically acceptable dose distribution using VMAT arc that is geometrically correlated with the 3DCRT beam arrangement. If it is not so, then appropriate modification should be done in the optimization technique to increase the optimizer efficiency. Furthermore, all optimizing engines should be able to accommodate an automatic flash margin to make the system efficient and reduce the planning time.
